# Brain regional homogeneity and function connectivity in attenuated psychosis syndrome —based on a resting state fMRI study

**DOI:** 10.1186/s12888-018-1954-x

**Published:** 2018-12-07

**Authors:** Xiangyun Long, Fei Liu, Nan Huang, Na Liu, Jie Zhang, Jing Chen, Ansi Qi, Xiaofeng Guan, Zheng Lu

**Affiliations:** 1 Department of Psychiatry, Shanghai Tongji Hospital, Tongji University School of Medicine, 389 Xin Cun Road, Shanghai, 200065 China; 2 Department of Psychiatry, Shanghai Mental Health Center, Shanghai Jiao Tong University School of Medicine, 600 Wan Ping Nan Road, Shanghai, 200030 China

**Keywords:** Attenuated psychosis syndrome, Resting-state functional magnetic resonance imaging, Regional homogeneity, Functional connectivity

## Abstract

**Background:**

By combining regional homogeneity (ReHo) and functional connectivity (FC) analyses, this study aimed to explore brain functional alterations in Attenuated Psychosis Syndrome (APS), which could provide complementary information for the neurophysiological indicators for schizophrenia (SZ) associated brain dysfunction.

**Methods:**

Twenty-one APS subjects and twenty healthy controls were enrolled in the data acquisition of demographics and clinical characteristics as well as structural and resting-state functional magnetic resonance imaging (rs-fMRI). ReHo analysis was conducted to determine the peak coordinate of the abnormal regional brain activity. Then, identified brain regions were considered as seed regions and were used to calculate FC between reginal brain voxels and whole brain voxels. Finally, potential correlations between imaging indices and clinical data were also explored.

**Results:**

Four APS and two HC subjects were excluded because the largest dynamic translation or rotation had exceeded 2 mm / 2°. Compared with healthy controls (HCs), APS subjects exhibited higher ReHo values in the right middle temporal gyrus (MTG) and lower ReHo values in the left middle frontal gyrus (MFG), left superior frontal gyrus (SFG), left postcentral gyrus (PoCG), and left superior frontal gyrus, medial (SFGmed). Considered these areas as seed regions, the APS subjects showed abnormal enhancement in functional brain connections, predominantly in the frontal and temporal lobes.

**Conclusions:**

We concluded that the APS subjects had spatially regional dysfunction and remoted synchronous dysfunction in the frontal and temporal lobes of the brain, and changes in ReHo and FC patterns may reveal the mechanism of brain dysfunctions and may serve as an imaging biomarker for the diagnosis and evaluation of SZ.

## Background

Seventy to ninety percent of schizophrenic patients would experience behavioral, verbal, and cognitive abnormalities before they reach the diagnosis of schizophrenia [1, 2]. Some researchers named this phase as Attenuated Psychosis Syndrome (APS), and this diagnostic term was also included in the section III of the Diagnostic and Statistical Manual of Mental Disorders, Fifth Edition (DSM-5) [3, 4]. The APS was also called “UHR (Ultra High Risk)” “ARMS (At Risk Mental State)” or “PRS (Psychosis Risk Syndrome)”, etc. [5], and it propelled scientists to research and develop preventions, identifications, and intervention programs for early psychosis. This definition helped us to screen out individuals with psychotic symptoms and need clinical interventions [6, 7]. Individuals with APS were considered to be highly at risk to psychosis: about 20–25% of them would convert to psychosis within 1 year and 30–35% within 2 years [8]. It is a well-accepted fact that even though schizophrenia (SZ) was classified as a severe mental illness, we don’t have much to serve as qualitative or quantitative biological indicators; its etiological research involved not only schizophrenic patients, but also included APS, first-degree relatives and other related groups. Previous studies were able to discover some symptomatology predictors in the APS group, including impairments in working memory, general intelligence, executive functions, verbal/visual memory, verbal fluency, attention and social cognition [9, 10] And since the functional magnetic resonance imaging (fMRI) was applied in this research, it became the excellent tool in exploring brain dysfunctions under mental symptoms.

Previous studies of APS neuroimaging were able to cover the resting-state functional magnetic resonance imaging (fMRI) and the task fMRI, such as discoveries in the prefrontal cortex (PFC) [8] and the white matter damage [11]. These structural anomalies propelled functional researches. Functional changes were often discussed along with changes in cognitive functions, language network dysfunctions [12],and activation anomalies in anterior cingulate cortex (ACC) [13] and bilateral PFC [14]. Social impairments had a negative correlation with anomalies in basal orbitofrontal cortex [15]. The combined results of functional and structural images seemed to be more precise in predicting APS subjects while comparing with results of unilateral image. Tognin used the method of multivariate machine learning to predict clinical symptoms and brain structure anomalies in APS subjects by gray matter volume and cortical thickness [16]. Benetti & Williams had compared genetic, cognitive and multi modal neuroimaging data to identify APS, first-episode psychosis (FEP) and healthy controls (HCs), and got 68.42% successful classification accuracies with structural magnetic resonance imaging and 65.79% with diffusion tensor neuroimaging in identifying APS subjects from healthy controls [17]. These results provided us with an inspiration, which is to analyze neuroimages of APS subjects.

It has been proved that organization functional brain networks develop from an anatomically local status to a more distributed structure [18]. In previous schizophrenia researches, a few guidelines had been established by studying regional and global brain function’s synchronization. Individuals identified as APS may be at a higher risk of developing schizophrenia than general population. Previous studies related to functional connectivity (FC) had also shown that this higher risk may have something to do with the unequal connection between ACC [13], ventral striatum [19], thalamus [20], bilateral frontal gyrus and other whole brain functional connections [21]. But the consistency of the study was not adequate enough due to recruitment of testers, different areas of interest, and analytical methods. And with the progress of methods and examination equipment, current results might point us a way to the disease pattern of psychosis. Some researches on APS made some progress in abnormal regional brain functions and brain synchronizations, which inspired us to embark on our study.

The aim of this article is to use function connectivity (FC) to seek brain function abnormities in APS subjects while compared to HCs. FC was operationally defined as statistical dependencies among distinct neurophysiological events, which can be measured by calculating correlations between time series of voxels or regional seeds, or both [22, 23]. In order to examine brain dysfunction areas preliminary, we used regional homogeneity (ReHo) to analyze brain function in resting state between groups, it can reflect the consistencies of local brain neurons activities based on calculating Kendall’s coefficient concordance (KCC) [24]. We introduced structured Interview for Prodromal Syndromes (SIPS) to evaluate participants for the attenuated psychosis syndrome, which has been proved to quantitatively assess the severity of prodromal symptoms for psychosis [25].

## Methods

### Participants

Twenty-one subjects with APS and 20 healthy controls (HCs) were recruited from studies of SZ early diagnosis between Feb 2014 and Dec 2014. All of our participants were outpatients from Shanghai Tongji Hospital and Shanghai Mental Health Center (SMHC) in China, and were all assessed by trained doctors. The ethical protocol was approved by Tongji hospital and SMHC, carried out in accordance with the Declaration of Helsinki. We ensured that all participants were informed of the study.

This study used the Chinese version SIPS, translated by Zheng [24], to evaluate the prodromal symptoms of APS subjects. SIPS is a structured interviewing tool based on SOPS (Scale of Psychosis-risk Symptoms), the full assessment takes about 1 h, and other scales including GAF (Global Assessment of Functioning scale), Schizotypal personality disorder checklist and a family history questionnaire [26, 27]. The purpose of using SIPS was to distinguish three prodromal syndromes defined by COPS (Criteria of Prodromal Syndromes) and psychosis onset defined by POPS (Presence of Psychotic Syndrome) [26, 27]. APS subjects must meet at least one category of COPS, and required to be 18–30 years old, Han nationality, right-handed, untreated with any antipsychotics and Wechsler Intelligence Scale’s value > 70.

HCs were recruited from volunteers and hospital staff and were matched to the participants’ age, gender, ethnicity, and educational level with the APS group. We used DSM-IV axis I disorder Structured Clinical Interview for Diagnostic (SCID assessment form) to exclude family history of mental illness or comorbidity.

We set the same exclusion criteria for both groups of subjects, including the inability to receive MRI, family history of mental illness, history of brain trauma or abnormal brain structures display by MRI, and history of alcohol or drug abuse. Demographic data, Positive and Negative Symptom Scale (PANSS) were used to assess their symptoms before performing the fMRI scan [28]. This process was carried out by two experienced psychiatrists who had the experience of 10 years at least from the SMHC.

### Image acquisition and preprocessing

SMHC undertook MRI scans of all subjects, A 3.0 T MRI scanner with an orthonormal coil (Siemens Magnetom *Verio 3.0*) was used to obtain a whole-brain T2*-weighted echo plane image. All subjects were asked to lie down with their eyes closed, stay awake, and keep their heads still. And then they accepted a consistent scanning process in a time of approximately 6 min and 40 s, included acquire high-resolution anatomical T1 images (parameters: TR (time to repetition) of 2300 ms, TE (time to echo) of 2960 ms, flip angle of 90°, FOV (field of view) of 256 mm in 256 × 256 matrix, slices thickness of 1.0 mm), and a total of 200 functional volumes (parameters: the same orientation as T1 images, in 3 mm voxel size with a TR of 2000 ms, TE of 30 ms, flip angle of 90° in 64 × 64 matrix size, and 220 mm FOV), continuous scanning 33 slices with slices thickness of 4.0 mm.

Preprocessing: The functional data preprocess complied with following steps:(1) Format conversion;(2) Remove the previous 10 unstable time points, leaving the remaining 190 time points;(3) Make slice timing using midpoint of time as reference;(4) Realign: set head motion via rigid-body alignment of each volume to the 17 scan, and four APS and two HC subjects were excluded because their largest dynamic rotation or translation had exceeded 2 mm / 2°;(5) Specialization of statistical parameters mapped echo-plane Image template in Montreal Institute of Neurology (MNI) Space used a nonlinear warping algorithm and the voxel were resampled to a 3 mm * 3 mm * 3 mm volume unit in a bounding box (− 90–126 -72;90 90,108);(6) Remove head movement parameters, white matter, cerebrospinal fluid signals and linear drift as covariates;(7) Filter (0.01 < f < 0.08 Hz). Used Resting-State fMRI Data Analysis Toolkit version 1.8(DPARSF *Advanced Edition*, http://rfmri.org/DPARSF) which was run in Matlab version 7.14(MathWorks *R2012a*) to conduct.

*FC analysis.*

ReHo calculations were performed using preprocessed images, and resulting images were smoothened with an isotropic Gaussian kernel of 4 mm full-width half- maximum (FWHM).

As mentioned above, we used ReHo analysis to seek the region of interest (ROI) which was calculated using DPARSF after preprocessing. Twenty-seven individual voxels were selected as a mass, using KCC (range 0 to 1) as indicators to measure ReHo value between each 27 individual voxels and 26 adjacent voxels. Each individual voxels ReHo was divided by the whole brain average ReHo, then the Gaussian smoothing was proceeded with Full Width Half Width (FWHW) 6 mm * 6 mm *6 mm to reduce space noise and error after space standards were produced. Then ReHo differences between the APS and HC group were analyzed with Two-Sample-T-test in Resting-State fMRI Data Analysis Toolkit (REST *version1.8*, http://pub.restfmri.net/) [29], and were further corrected by monte-carlo simulation (Alphasim correction).

FC calculation was conducted with REST. The coordinate of the center of seed regions was based on the inter-group analysis of ReHo, and its radius was 6 mm. Correlation analysis of time course between the spherical seed area and the whole brain voxel was conducted on each subject. Finally, Fisher’s r-to-z transformation was applied to improve the normality of the ReHo and FC maps.

### Statistical analysis

Clinical data and neuropsychological test scores of APS and HC groups were compared using the SPSS software (version22.0,). Intra-group differences of ReHo and FC were analyzed with one-sample t-test, and inter-group differences between APS and HCs were analyzed with two-sample t-test in REST. The following results were corrected using Monte-Carlo simulation in rest with the program Alphasim, set the statistical threshold of *p* < 0.001 (Alphasim correction:cluster *p* < 0.001, cluster size >13voxels, full width at half maximum = 6 mm, number of Monte Carlo simulations = 1000, cluster connection radius: rmm = 5.00) to detect and compare brain regions that survived.

## Results

### Demographics and clinical characteristics of the APS group and the HC group

Eventually, after excluding subjects with excessive head movements, 17 APS subjects and 18 HC subjects entered the statistical analysis stage. Compared with the control group, the APS group had no significant difference between age, education, gender, and body mass index (BMI). (Complete information in Table 1).

**Table 1:** Demographics and Clinical Characteristics of the APS Group and the HC Group ($\overline{x}\pm s$)

	APS(*n* = 17)	HC(*n* = 18)		*t*/X^2^	*p*
Age (years)	22.94 ± 3.39	25.22 ± 3.68		− 1.987	.054
Education level (years)	13.18 ± 1.84	12.78 ± 2.26		.565	.576
BMI (kg/m2)	20.58 ± 1.88	21.15 ± 1.70		−.953	.347
Gender, Male(%)	8 (47.06)	8 (44.44)		.024	.573
PANSS					
Positive	13.18 ± 1.19				
Negative	12.18 ± 3.59				
General Pychopathology	24.47 ± 2.37				
GAF score	57.05 ± 3.63				
Family History of Psychosis Disorder, n (%)	5 (27.77)				
Primary SIPS-defined prodromal status, n (%)					
Attenuated Positive Symptom State (APSS)	17 (100)				

$\overline{x}$ Mean value, *s* Standard deviation, *APS* Attenuated psychosis syndrome, *HC* Healthy controls

### Regional homogeneity analysis

**Fig. 1:**
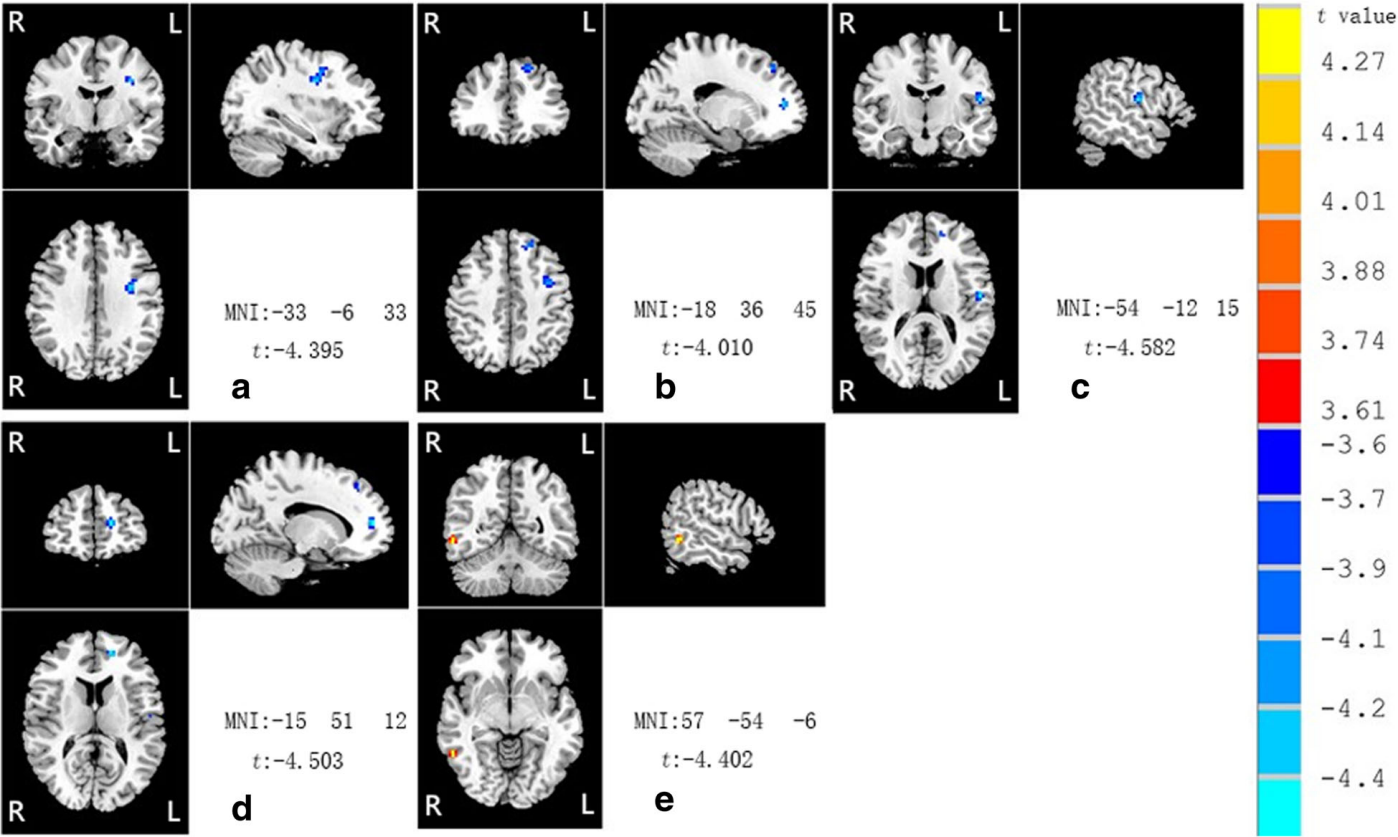
Compared with control group, APS group exhibited higher ReHo values in the right middle temporal gyrus (MTG) and lower ReHo values in the left middle frontal gyrus (MFG), left superior frontal gyrus (SFG), left postcentral gyrus (PoCG), left superior frontal gyrus, medial (SFGmed) (**a** left MFG; **b** left SFG and left SFGmed; **c** left PoCG and left SFGmed; **d** left SFGmed and left SFG; **e** right MTG)

Compared with control group, APS group exhibited significantly higher ReHo values in the right middle temporal gyrus (MTG) and lower ReHo values in the left superior frontal gyrus (SFG), left postcentral gyrus (PoCG), left middle frontal gyrus (MFG), left superior frontal gyrus, medial (SFGmed). (Complete information in Table 2 and Fig. 1).

**Table 2:** Brain regions with different ReHo values between APS and HC groups

Brain regions	MNI:peak voxel			Cluster (voxels)	Mean ReHo value(±SD)		*t* value
	x	y	z		APS	HC	
L Middle frontal gyrus	−33	− 6	33	61	0.85 (0.18)	1.27 (0.32)	−4.395
L Superior frontal gyrus	−18	36	45	20	0.90 (0.16)	1.17 (0.20)	−4.010
L Postcentral gyrus	−54	−12	15	19	0.95 (0.20)	1.24 (0.27)	−4.582
L Superior medial frontal gyrus	−15	51	12	17	0.84 (0.30)	1.30 (0.32)	−4.503
R Middle temporal gyrus	57	−54	−6	13	1.07 (0.27)	0.76 (0.13)	4.402

*APS* Attenuated psychosis syndrome, *HC* Healthy controls, *L* Left, *R* Right*, MNI* Montreal neurological institute, *ReHo* Regional homogeneity, voxel *p* < 0.001 (AlphaSim correction, cluster *p* < 0.001, cluster size > 13voxels)

### Functional connectivity analysis

**Fig. 2:**
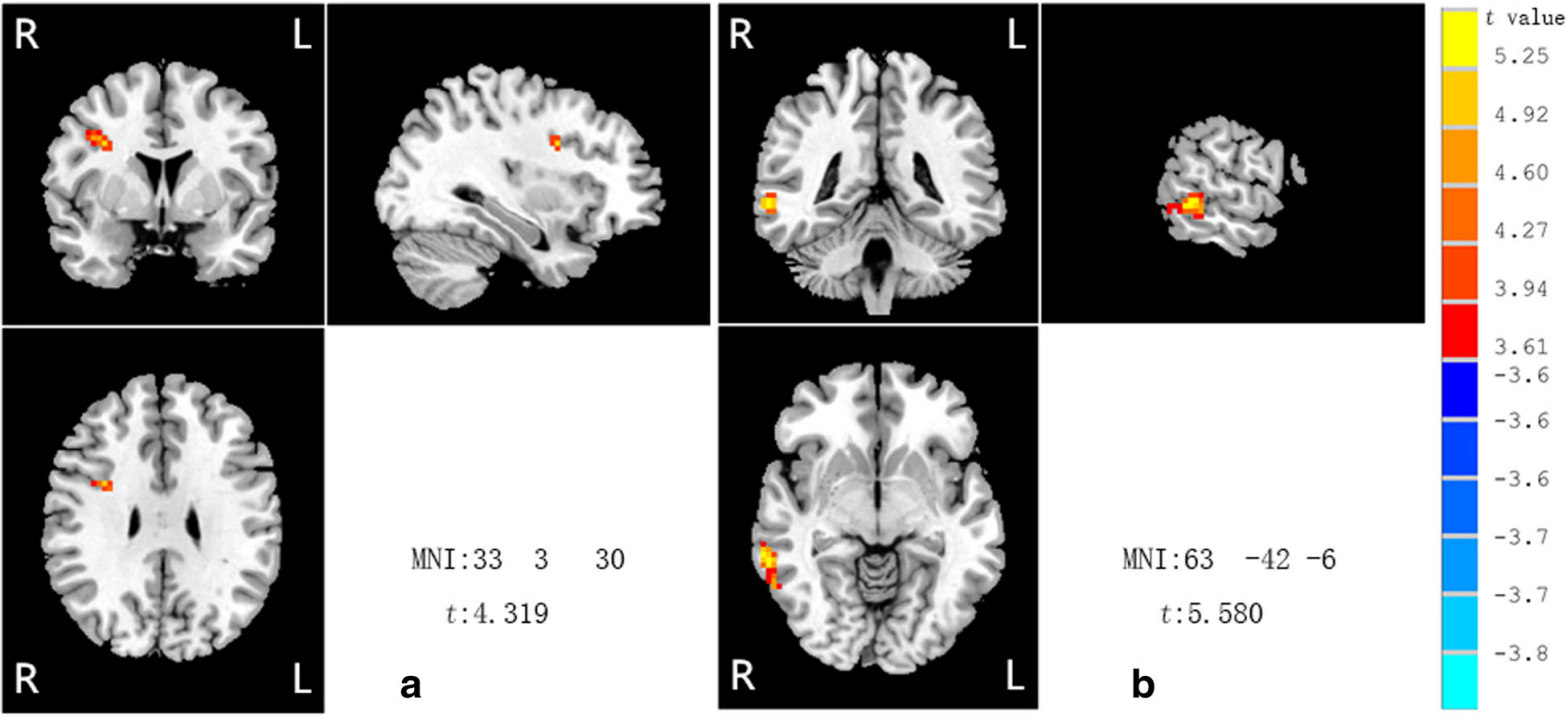
Compared with the control group, APS group exhibited increased FC between left MFG and right inferior frontal gyrus (IFG), increased FC between right MTG and right IFG (**a** seed area: left MFG; **b** seed area: right MTG.)

Compared with the control group, FC between left MFG and right inferior frontal gyrus (IFG), and FC between right MTG to right IFG were increased in the APS group. no positive results were found in the use of other seed points including the left SFG, left PoCG and left SFGmed. (complete information in Table 3 and Fig. 2). Considering with ReHo results, the APS subjects showed abnormally enhanced functional brain connections, predominantly in the frontal and temporal lobes.

**Table 3:** Brain regions with different FC between APS and HC groups

Seed area	Area with altered functional connectivity	MNI:peak voxel			Cluster (voxels)	*t* value
		x	y	z		
L Middle Frontal gyrus	R Inferior frontal gyrus	33	3	30	21	4.319
L Superior Frontal gyrus	No significant results					
L Postcentral gyrus	No significant results					
L Superior medial frontal gyrus	No significant results					
R Middle temporal gyrus	R Inferior temporal gyrus/R Middle temporal gyrus	63	−42	−6	63	5.580

*APS* Attenuated psychosis syndrome, *HC* Healthy controls, *L* Left, *R* Right, *MNI* Montreal neurological institute, *FC* Functional connectivity, voxel *p* < 0.001 (AlphaSim correction, cluster *p* < 0.001, cluster size > 13voxels)

## Discussion

Many previous studies have shown the superiority of using fMRI to detect brain dysfunctions among schizophrenic patients, the research’s direction had transferred from structural isomerization to single regional dysfunction and now focused on functional connection differences in multiple regions, which refers to the correlation of simultaneous neural activities between the anatomically separated brain regions [30]. Based on the hypothesis that separated brain regions worked together in a stable functional network and maintained a high level sustained spontaneous neuronal activity in resting states, functional connectivity methods were used to explore the pattern and abnormality of inter-regional functions [31]. Along with the theory above, Some functional sub-networks had been proposed and validated, and these results pushed the fMRI research to be one of the hotspots of mental illness research [32].

By analyzing different functional connectivity between the frequency range of 0.06–0.125 Hz among 15 HCs and 12 SZ patients, the resting-state fMRI study found that the functional connectivity of SZ patients were significantly reduced but the diversity increased [33]. By utilizing ICA topological measures to analyze the rest-state functional network connections in 19 patients, Qingbao Yu discovered patterns of functional connectivity in some regions including frontal, parietal, occipital and cerebellar in SZ group had changed. [19]. In the intra cortical connections, many previous researches focused on abnormal connections of the prefrontal cortex.

Compared to the HCs, APS subjects in this article showed a significantly lower ReHo in left SFG, SFGmed, PoCG, MFG, and a higher ReHo in the right MTG during the resting state. Further FC analysis showed aberrant functional connectivity in the temporal gyrus and the frontal lobe. These abnormalities suggested that it is highly possible that the APS subjects suffered from the disease, which could lead to brain dysfunction from local to global scales. Results of our study were mainly focused on the abnormalities of the frontal and temporal lobes and will be discussed later in the article.

The frontal lobe was an important brain region anatomically and functionally, which sub serve human decision-making and executive control [34]. Previous studies had divided it into sub-regions based on location and function, including the anterior cingulate, orbital, posterior frontal lobe boundary, etc. A study of neuroanatomical abnormalities in PRS patients found that PRS subjects whom later developed psychosis had lesser grey matter in their inferior frontal gyrus [35], similar findings were also found in SZ subjects [36]. The right inferior frontal gyrus in PRS subjects showed a greater regional functional synchronization in other fMRI researches and showed an increase of function connectivity in our study [37, 38] DTI and task-related fMRI studies found abnormal functional connections in the left and right IFG [39, 40]. Some studies on schizophrenic patients with drug intervention also points to the variation on structure and function of the frontal lobes [41, 42]. On the whole, our findings on the frontal lobe were consistent with previous studies, which is the importance of frontal lobe in the pathogenesis of SZ.

Previous studies had found that the middle temporal gyrus (MTG) was involved in the default mode (DMN), which was crucial in many cognitive domains such as language processing and deductive reasoning. A cross-sectional comparison found that in the right medial temporal area, the volume of gray matter was reduced in PRS subjects who had developed psychosis [43]. And not only PRS subjects had a cortical thinning influenced volumetric reductions of the middle temporal gyrus [35], the same reductions can also be observed among First-Episode and Chronic SZ patients studies [44–46]. A study of long-term follow-up of school-age mental illness found that in participates whom developed to SZ, the abnormal decrease of MTG volume preceded the appearance of mental symptoms. [44]. Facial emotion recognition task related MRI also found volume and function changes in temporal lobe in patients with SZ, which may be associated with their impaired facial expression recognition [47]. Studies on brain function differences have confirmed that the temporal gyrus contains multiple sub regions, which was consistent with the results of ReHo and functional synchronization abnormalities in the right temporal gyrus in this study.

There are some restrictions need to be resolved in our future studies. First, we just included a limit number of samples, Most APS populations were children or adolescents, to research on them would have to face multiple limitations such as ethics, schools, families, and so on. And to prepare them for MRI was too difficult and time-consuming, future researches should be focused on improving research projects and designing a more reasonable collection method to overcome these difficulties. Second, patients’ compliance with the study wasn’t good enough, and their families and schools lacked attention, so we were not able to obtain sufficient follow-up data. Third, for the tools we used in the research, such as SIPS, ReHo, FC method, etc. These templates may not be fully applicable to the Chinese population.

## Conclusions

Our re-fMRI study on the APS population showed that there might be abnormal regional and global brain function synchronization at the resting state. These findings complemented our predecessor’s research and the aim were both to explore the pathogenesis of schizophrenia. We have some train of thought: First, the prodromal phase should be a continuous state; not only the appearance of clinical symptoms, but also the gradual decline of the patient’s functions can be served as a starting point of research; Second, it’s still unclear whether the abnormal brain function we found in the APS population can directly lead to the appearance of its clinical symptoms; Third, if a model is used to integrate anomaly indicators in multiple dimensions such as brain functional imaging, electroencephalography, physiology, and symptomatology assessments for patients with SZ, can we obtain a more consistent result?

## Abbreviations

ACC: Anterior cingulate cortex
APS: Attenuated Psychosis Syndrome
ARMS: At risk mental state
COPS: Criteria of Prodromal Syndromes
DSM-5: Diagnostic and Statistical Manual of Mental Disorders, Fifth Edition
DSM-IV: Diagnostic and Statistical Manual of Mental Disorders, Fouth Edition
DTI: Diffusion tensorimaging
FC: Functional connectivity
FEP: First-episode psychosis
FOV: Field of view
FWHW: Full-width half- maximum
GAF: Global Assessment of Functioning scale
HC: Healthy control
KCC: Kendall’s coefficient concordance
MFG: Middle frontal gyrus
MNI: Montreal Neurological Institute
MTG: Middle temporal gyrus
PANSS: Positive and Negative Symptom Scale
PFC: Prefrontal cortex
PoCG: Postcentral gyrus
POPS: Presence of Psychotic Syndrome
PRS: Psychosis Risk Syndrome
ReHo: Regional homogeneity
ROI: Region of interest
rs-fMRI: Resting-state functional magnetic resonance imaging
SCID: Structured Clinical Interview for Diagnostic
SFG: Superior frontal gyrus
SFGmed: Superior frontal gyrus, medial
SIPS: Structured Interview for Prodromal Syndrome
SMHC: Shanghai Mental Health Center
SOPS: Scale of Psychosis-risk Symptoms
TE: Time to echo
TR: Repetition
UHR: Ultra High Risk
